# Age-dependent modulation of bone metabolism in zebrafish scales as new model of male osteoporosis in lower vertebrates

**DOI:** 10.1007/s11357-020-00267-0

**Published:** 2020-09-30

**Authors:** Marta Carnovali, Giuseppe Banfi, Massimo Mariotti

**Affiliations:** 1 IRCCS Istituto Ortopedico Galeazzi, Milan, Italy; 2 Vita-Salute San Raffaele University, Milan, Italy; 3 Department of Biomedical, Surgical and Dental Sciences, University of Milan, Milan, Italy

**Keywords:** Zebrafish, Scale, Bone, Aging, Osteoporosis

## Abstract

After middle age, in human bone, the resorption usually exceeds formation resulting in bone loss and increased risk of fractures in the aged population. Only few in vivo models in higher vertebrates are available for pathogenic and therapeutic studies about bone aging. Among these, male *Danio rerio* (zebrafish) can be successfully used as low vertebrate model to study degenerative alterations that affect the skeleton during aging, reducing the role of sex hormones.

In this paper, we investigated the early bone aging mechanisms in male zebrafish (3, 6, 9 months old) scales evaluating the physiological changes and the effects of prednisolone, a pro-osteoporotic drug.

The results evidentiated an age-dependent reduction of the mineralization rate in the fish scales, as highlighted by growing circle measurements. Indeed, the osteoblastic ALP activity at the matrix deposition site was found progressively downregulated.

The higher TRAP activity was found in 63% of 9-month-old fish scales associated with resorption lacunae along the scale border. Gene expression analysis evidentiated that an increase of the *tnfrsf1b* (homolog of human *rank*) in aging scales may be responsible for resorption stimulation.

Interestingly, prednisolone inhibited the physiological growth of the scale and induced in aged scales a more significant bone resorption compared with untreated fish (3.8% vs 1.02%). Bone markers analysis shown a significant reduction of ALP/TRAP ratio due to a prednisolone-dependent stimulation of *tnfsf11* (homolog of human *rankl)* in scales of older fish.

The results evidentiated for the first time the presence of a senile male osteoporosis in lower vertebrate. This new model could be helpful to identify the early mechanisms of bone aging and new therapeutic strategies to prevent age-related bone alterations in humans.

## Introduction

After middle age (35–40 years), in humans, bone resorption usually exceeds its formation resulting in a bone loss condition, called primary osteoporosis, characterized by increased risk of fractures in aged population [52]. The osteoporosis in elderly is related to oxidative stress, inflammation and cellular senescence [16, 35].

Several studies reported that the mineralization ability of osteoblasts was mainly reduced in aging with a progressive reduction of bone growth [7]. Osteoblast-specific genes, such as Runt-related transcription factor 2 (Runx-2), show the same expression profile in mesenchymal stem cells (MSCs) as well as in peripheral blood mononuclear cells (PBMCs), and they are highly correlated with bone mineral density (BMD) and age in men and women [55].

On the other hand, bone catabolic activity has the tendency to increase with age. In particular, in vitro osteoclastogenesis increases with age due to a modulation of regulatory factors expressed in the bone marrow like macrophage-colony stimulating factor (M-CSF), osteoprotegerin (OPG), receptor activator of NF-κB (RANK) and receptor activator of NF-κB ligand (RANKL) [8].

Primary osteoporosis should be distinguished in female post-menopausal form and male form, depending of differences between the sexes in hormonal physiology and its consequences on bone tissue [43]. The drop in oestrogen levels that characterizes the menopause in female contrasts with the gradual downregulation of sex hormones (andropause) in aging men [19].

Since hormonal factors have a dominant role in female osteoporosis, in vivo experimental models on male animals have been used to investigate the characteristics and the basic molecular mechanisms of age-related bone loss [35, 51]. Nevertheless, the complexity of the cross-talk systems and metabolic and physical-chemical regulations of higher vertebrate makes it difficult to dissect the molecular and cellular mechanisms driving the early phase of bone aging. A simpler animal model in lower vertebrate could be very helpful to extend the knowledge in this specific field of study.

The adult zebrafish is gaining importance as innovative and readily available resource for studying skeletal system [3, 14, 50] and bone metabolism both at cellular and molecular level. In particular, the scale represents a unique anatomical feature thanks to the presence of a bone cell and mineralized matrix similar to human lamellar bone and with undoubted advantages like transparency and easy handling [5]. These characteristics allows using vital dyes to visualize and easily measure the mineral matrix deposition and resorption. The same approach cannot be applied to internal bones.

The basal mechanisms of bone loss during aging can be better evidentiated in male fish because of non-relevant modulation of oestrogen levels during aging [46] and because of a testosterone level comparable with female [37].

Zebrafish bone tissues are characterized by age-related changes in their structure, organization and composition. For example, old zebrafish (over 1 year after fertilization) shows vertebral column deformity with interesting similarities with degenerative joint disease in humans [21], while first alterations in bone microarchitecture can be evidentiated earlier (from 6 months after fertilization) in vertebral bodies [6, 38].

These data indicated that zebrafish is a powerful model to study the degenerative changes in the axial skeleton during aging, from early events to the clinical evidences in older fish. Nevertheless, few data have been produced about the early mechanisms of bone aging and their effects on cell behaviour, matrix turnover and gene regulation [6, 38].

In this work, we investigated early bone aging mechanisms in male zebrafish using scales as read-out system and analysing the physiological changes and the effects of prednisolone, as pro-osteoporotic stimulus, in fish of different ages. This model will help to define early basic mechanisms of age-related bone osteopenia and which therapeutic strategy or agent could be used to modulate bone alterations in humans.

## Methods

### Animals and treatments

Zebrafish of the AB strain were maintained in ZEBTEC© Bench Top System (Tecniplast, Italy) under standard conditions [53] at 28 °C with a photoperiod of 14 h light and 10 h dark. This experimentation has used male fish aged 3, 6 and 9 months, using a total of 126 fish. Fish of the 3 different ages comes from different population since our preliminary studies demonstrated that there is no variability between different cohorts of fish of the same age (same length and weight, data not shown). Treatments have been performed maintaining fish in E3 medium (5 mM NaCl, 0.17 mM KCl, 0.33 mM CaCl_2_, 0.33 mM MgSO_4_) at 28 °C. Zebrafish osteoporotic model has been induced treating singularly the fish with 80 μM prednisolone (1-dehydrohydrocortisone, PN, Sigma Aldrich) for 14 days [41]. PN has been initially dissolved in dimethyl sulfoxide (DMSO) stock solution, then diluted in E3 medium to create the final treatment solution. Treatment has been performed maintaining fish in 250 ml of E3 medium with 80 μM PN changing the solution every 48 h. Fish have been fed 3 times each day using standard commercial fish food (Vipagran, Sera).

### Sample collections

Blood collection has been performed according to Eames’s previously published protocol [53]. Briefly, MS-222 (tricaine; Sigma) was used at 0.02% to induce anaesthesia, and then fish were decapitated by cutting through the pectoral girdle with scalpel. Whole blood was collected using a heparinized 100-mL microcapillary tube (Sarstedt) adjacent to the severed heart. Scales have been carefully removed from both sides of fish body in the ventral area, under light stereomicroscope (Olympus SZX-ZB7) using Dumont® Steel Forceps (Sigma Aldrich) then processed differently depending on the analysis to be performed as described below.

### Double bone matrix vital staining

To visualize and quantify the new bone matrix deposited on the scale as new mineralized growing ring, fish have been double stained using two dyes specific for the mineralized matrix, following our previously published protocol [41]. Briefly, at the beginning of the experiments, fish have been live stained with a 0.005% Alizarin Red S (ARS, Sigma Aldrich) E3 solution overnight in the dark at 28 °C. After 2 weeks, fish have been live stained overnight in the dark with a 0.005% calcein (Bis[N,N-bis(carboxymethyl)aminomethyl]fluorescein, Sigma Aldrich) E3 solution. After repeated washes, scales have been collected as previously described and fixed in 3.5% formaldehyde 0.1 M sodium phosphate buffer solution, then scales have been analysed using a fluorescence microscope (Olympus SZX-ZB7) equipped with a Discovery CH30 camera (TiEsseLab). Scale growing ring and scale area have been measured using ISC Capture Software (TiEsseLab).

### Biochemical alkaline phosphatase (ALP) and tartrate-resistant acid phosphatase (TRAP) activity assays

Explanted scales have been fixed using a 10% formalin 0.05 cacodylate buffer (pH 7.4) solution, and then biochemical TRAP activity has been evaluated using a test published by Perrson et al. [42], while biochemical ALP activity was evaluated according to the previously published method [39]. The absorbance of the resulting solutions has been read at 405 nm using a spectrophotometer (iMarkTM Microplate Reader, Bio-Rad) and converted into the amount of produced para-nitrophenol (pNP) using a standard curve for pNP. The resulting values have been normalized for scale area values, then used to calculate ALP/TRAP ratio.

### Histological ALP and TRAP activity assays

To perform the histological ALP assay, scales have been fixed with a 10% formalin 0.05 cacodylate buffer (pH 7.4) solution, rinsed in PBS and then exposed to BCIP®/NBT liquid substrate (Sigma Aldrich) according to manufacturer’s protocol. Histological TRAP activity assay has been performed using Leukocytes Acid Phosphatase (TRAP) Detection Kit according to the manufacturer’s protocol (Sigma Aldrich). Stained scales have been analysed using a stereo-microscope (Olympus SZX-ZB7) equipped with a Discovery CH30 camera (TiEsseLab) to identify TRAP-positive scales and quantify the resorbing area.

### Gene expression analysis

Total RNA has been isolated from the scales using Euro-GOLD Total RNA Kit (Euroclone), and the complementary DNA (cDNA) was obtained using High Capacity cDNA Reverse Transcription Kit (Applied Biosystems). Real-time PCR analysis has been performed with StepOne™ Real-Time PCR System (Applied Biosystems) using FluoCycle II™ SYBR® Master Mix (Euroclone). The expression analysis was focused on bone-specific genes such as tumour necrosis factor receptor superfamily member 11b (*tnfrsf11b*, fish homolog of human *osteoprotegerin*, OPG), tumour necrosis factor receptor superfamily member 1b (*tnfrsf1b*, fish homolog of human *rank*) and tumour necrosis factor ligand superfamily member 11 (*tnfsfl1*, fish homolog of human *rankl*).

Each PCR analysis has been performed in triplicate. The gene-specific primers have been previously published [26, 48], and the sequences were OPG_F 5′- GGCGTCTGAAGAAACCTCTG-3′ OPG_R 5′- GCAGGATTGGGATGCAGTAT-3′; RANK_F 5′-AAGTGGACAGATTGTAAAGCTAT-3′ RANK_R 5′-GCCACCTGATGAGGTTTCAGCAC-3′; RANKL_F 5′- TAGTGTGGCGATTCTGTTGC-3′ RANKL_R 5′- ATTGGAAGGTGAGCTGATGG-3′; β-ACTIN_F 5′- CGAGCGTGGCTACAGCTTCA-3′ β-ACTIN_R 5′- GACCGTCAGGCAGCTCATAG-3′. The amplification conditions have been previously published [26] and consist of 3 min of initial denaturation at 95 °C followed by 40 cycles of denaturation at 95 °C for 30 s, annealing and extension at 60 °C for 40 s with a single cycle for dissociation curve analysis. Analysis have been performed using the Ct method with β-actin as the endogenous control. The average ΔCt value was calculated by subtracting the control ΔCt from the treated ΔCt and the relative quantity of mRNA was calculated as 2^-ΔΔCt^. Real-time PCR results have been used to calculate *tnfsfl1*/*tnfrsf11b* ratio [44].

### PTH blood analysis

Zebrafish blood has been collected and used to quantify parathyroid hormone (parathormone, PTH) by PTH ELISA test according to the manufacturer’s protocol (Fish Parathormone Intact ELISA kit, MybioSource). The absorbance has been read with a spectrophotometer (iMarkTM Microplate Reader, Bio-Rad).

### Statistics

Fish of 3 different ages (3, 6 and 9 months) have been used to perform bone tissue analysis on the scales. Each experiment has been performed using 14 fish of each age (42 fish in total), treating 7 fish with prednisolone and using 7 fish as control. The whole experiment has been repeated 3 times using a total of 126 fish.

Biochemical analysis of ALP and TRAP activity have been performed using 20 scales for fish for each analysis, while histological assays have been performed on 50 scales. All the remaining scales were used to perform the gene expression analysis. The blood obtained from 7 fish has been pooled and used to perform PTH ELISA assay. The qPCR experiments of bone marker genes have been repeated three times with comparable results. All the collected data have been analysed using the one-way analysis of variance (ANOVA) followed by Bonferroni test for multiple comparisons, and results have been expressed as mean ± standard deviation (SD) vs control. All these tests have been repeated 3 times with comparable results, and the significance values have been set at less than *p* < 0.05 (*), *p* < 0.01 (**) and *p* < 0.001 (***).

## Results

### Zebrafish scale growing circle thickness is age-dependent

**Fig. 1:**
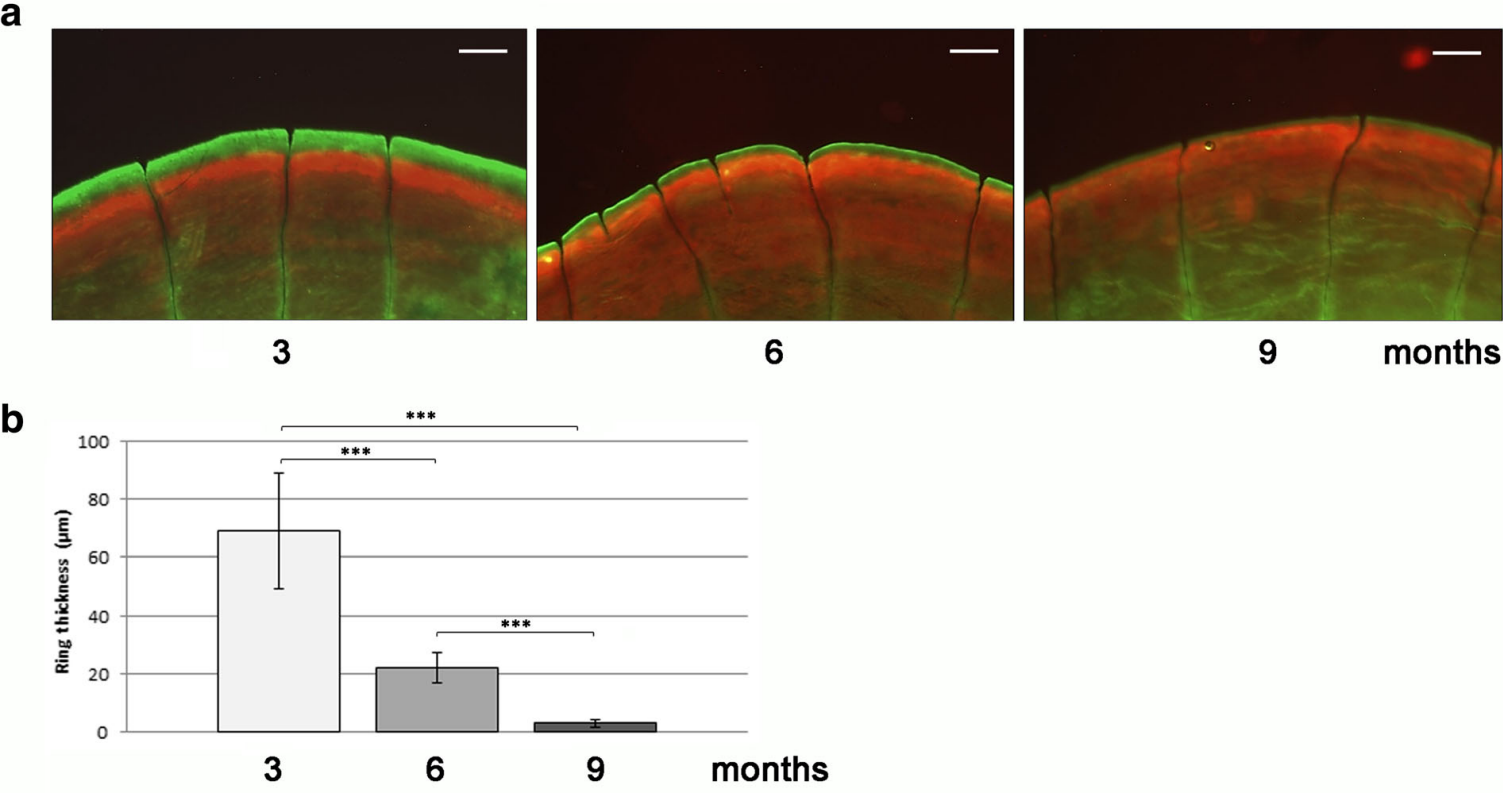
**a** Alizarin red S/calcein live double staining in scales from fish of different ages (scale bar = 0.1 mm). Three-month-old fish showed a significant growth rate identified as thickness of the new deposited ring (green fluorescence). In 6-month-old fish scales, the thickness of new ring was found reduced, whereas 9-month-old fish did not show any significant deposition. **b** Quantification of the ring thickness in scales from 3-, 6- and 9-month-old fish (3 months vs 6 months, *p* < 0.001; 3 months vs 9 months, *p* < 0.001; 6 months vs 9 months, *p* < 0.001). The mineralization rate was inversely proportional to the age of the fish

Double vital staining with alizarin red S-calcein has been used to calculate the mineralization rate in the scale as thickness of the new ring deposited [41]. The scales of 3-month-old fish (young adult) formed a thick growing ring as expression of intense matrix deposition. In 6-month-old fish (adult fish) scales, the physiological thickness of new ring was found reduced, whereas 9-month-old fish (old fish) did not show a significant mineralized matrix deposition (Fig. 1a). The quantification of the ring thickness confirmed that the mineralization rate is inversely proportional to the age of the fish since 6 months-old fish ring thickness is − 68% of the 3 month-old ring and the thickness of the 9 month-old fish ring is − 95% of the 3 month-old ring (Fig. 1b; 3 months vs 6 months, *p* < 0.001; 3 months vs 9 months, *p* < 0.001; 6 months vs 9 months, *p* < 0.001).

### Anabolic bone activity is modulated by age in zebrafish scale

**Fig. 2:**
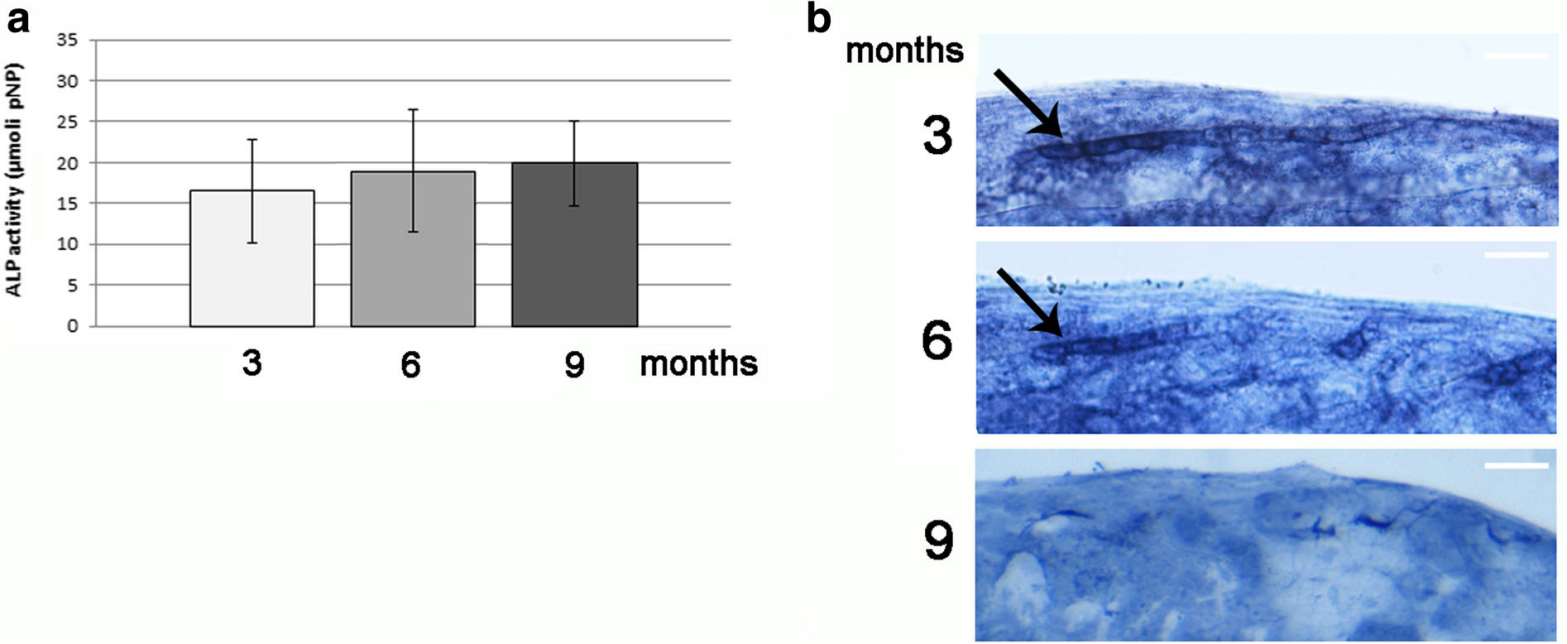
**a** Anabolic activity on zebrafish scales measured by ALP biochemical assay normalized for scale area. No modulation of ALP activity was observed in 3-, 6- and 9-month-old fish. **b** Histochemical staining for ALP activity on aging scales (scale bar = 0.05 mm). A strong ALP activity has been detected in matrix-deposing cells (black arrow) in 3-month-old fish. The signal has become slightly downregulated in 6-month-old fish (black arrow) and disappeared in 9-month-old fish

Alkaline phosphatase (ALP) is a very well-known marker of bone anabolic activity. Explanted scales from 3-, 6- and 9-month-old fish have been used to measure ALP activity through a biochemical assay. No significant modulation of ALP activity in the scales was found associated with the aging of the fish (Fig. 2a).

As demonstrated in literature, the cells responsible for the mineralized matrix deposition in the scales are characterized by intense ALP activity [40]. The histochemical staining for ALP activity has been performed on zebrafish scales from 3-, 6- and 9-month-old fish to highlight the differences in osteoblastic activity at the primary deposition site. The matrix-deposing cells appeared strongly positive for ALP activity in 3-month-old fish, whereas the signal decreased in 6-month-old fish (Fig. 2b). Later, in 9-month-old fish, no differences were detected in ALP activity at deposition site (Fig. 2b), suggesting an arrest of the bone production process.

### Catabolic bone activity is modulated by age in zebrafish scales

**Fig. 3:**
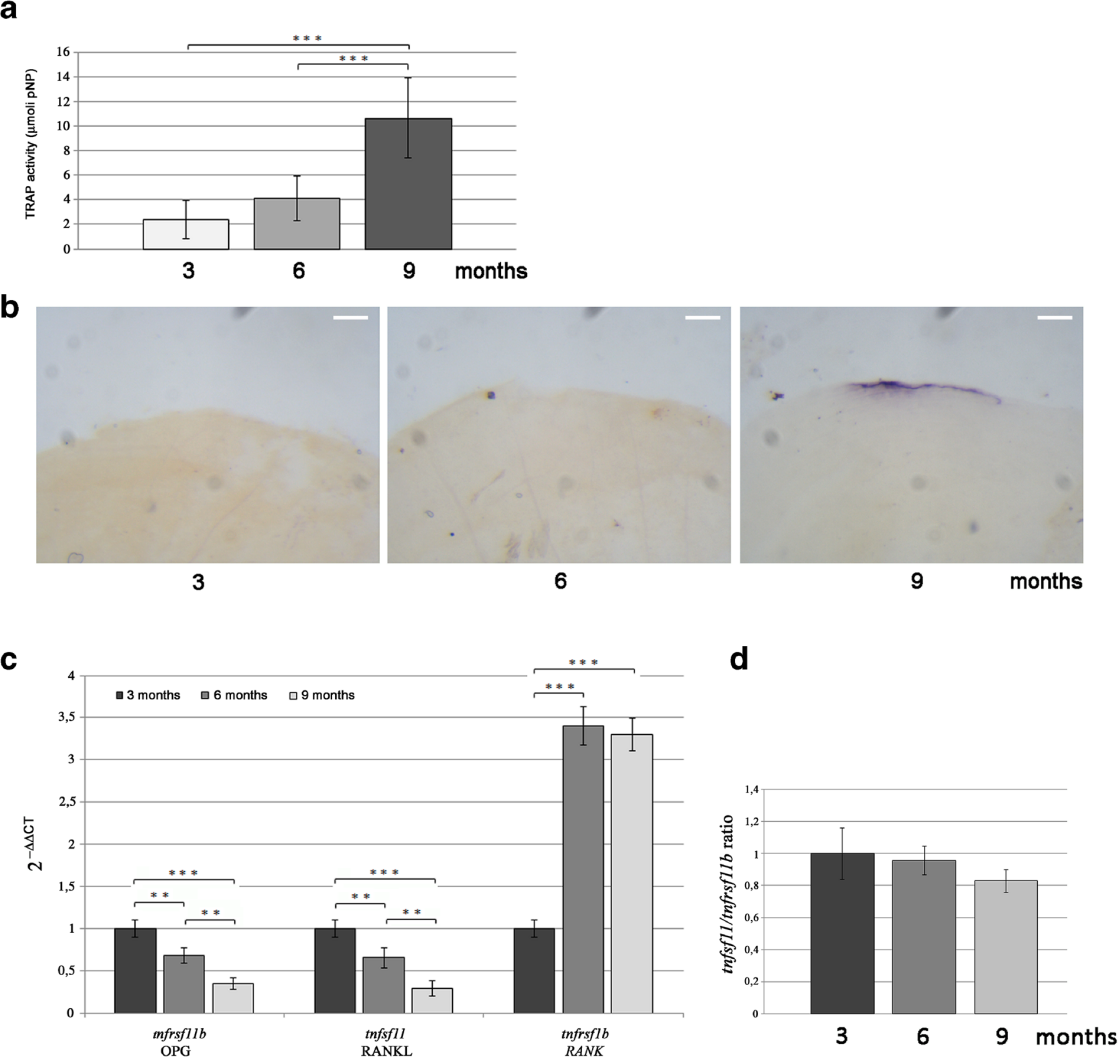
**a** TRAP, measured by biochemical assay and normalized for scale area, has indicated a progressive increase of the activity in 3-, 6- and 9-month-old fish (3 months vs 9 months, *p* < 0.001; 6 months vs 9 months, *p* < 0.001). **b** Histochemical staining for TRAP activity on aging scales (scale bar = 0.1 mm). TRAP-positive signal has been detected along scale border of 9-month-old fish surrounding a bone resorption area. **c** Gene expression analysis on aging scales. Real-time PCR for bone-specific genes has shown an increase of *tnfrsf1b* (homolog of human *rank*) expression in scales from 6- and 9-month-old fish and an age-dependent downregulation of *tnfsf11* and *tnfrsf11b* (homolog of human *rankl* and *opg*) (OPG: 3 months vs 6 months, *p* < 0.01; 3 months vs 9 months, *p* < 0.001; 6 months vs 9 months, *p* < 0.01. RANKL: 3 months vs 6 months, *p* < 0.01; 3 months vs 9 months, *p* < 0.001; 6 months vs 9 months, *p* < 0.01. RANK: 3 months vs 6 months, *p* < 0.001; 3 months vs 9 months, *p* < 0.001). **d** Calculated *tnfsf11*/*tnfrsf11b* (*rankl/opg*) ratio was found unchanged during aging

The biochemical analysis of tartrate-resistant acid phosphatase (TRAP) activity on zebrafish scales from 3-, 6- and 9-month-old fish has indicated a significant increase related to the age of fish since 6-month-old TRAP is increased by 72% and 9-month-old TRAP is increased by 345% compared with 3-month-old fish TRAP (Fig. 3a; TRAP: 3 months vs 9 months, *p* < 0.001; 6 months vs 9 months, *p* < 0.001). In order to visualize the active osteoclasts in the scales, a histochemical staining has been performed on the same fish identifying TRAP-positive signal only in 9-month-old fish associated with small resorption lacunae along the border of the scale (Fig. 3b). The calculated area of the bone loss corresponded to an average of 1.02% of the scale surface (not shown). Biochemical TRAP analysis (Fig. 3a) detected a low TRAP activity in scales of 3 and 6 months old fish, but it is so low that it can be considered as background noise as confirmed by the histological analysis.

Gene expression analysis performed by real-time PCR has shown an increase of *tnfrsf1b* (homolog of human *rank*) in scales from 6- and 9-month-old fish and an age-dependent downregulation of *tnfsf11* and *tnfrsf11b* (homolog of human *rankl* and *opg*) (Fig. 3c OPG: 3 months vs 6 months, *p* < 0.01; 3 months vs 9 months, *p* < 0.001; 6 months vs 9 months, *p* < 0.01. RANKL: 3 months vs 6 months, *p* < 0.01; 3 months vs 9 months, *p* < 0.001; 6 months vs 9 months, *p* < 0.01. RANK: 3 months vs 6 months, *p* < 0.001; 3 months vs 9 months, *p* < 0.001). The *tnfsf11*/*tnfrsf11b* (*rankl/opg*) ratio, known to be involved in the regulation of bone resorption, was found unchanged by comparing the ages (Fig. 3d).

### The aging modulates bone turnover regulatory pathways

**Fig. 4:**
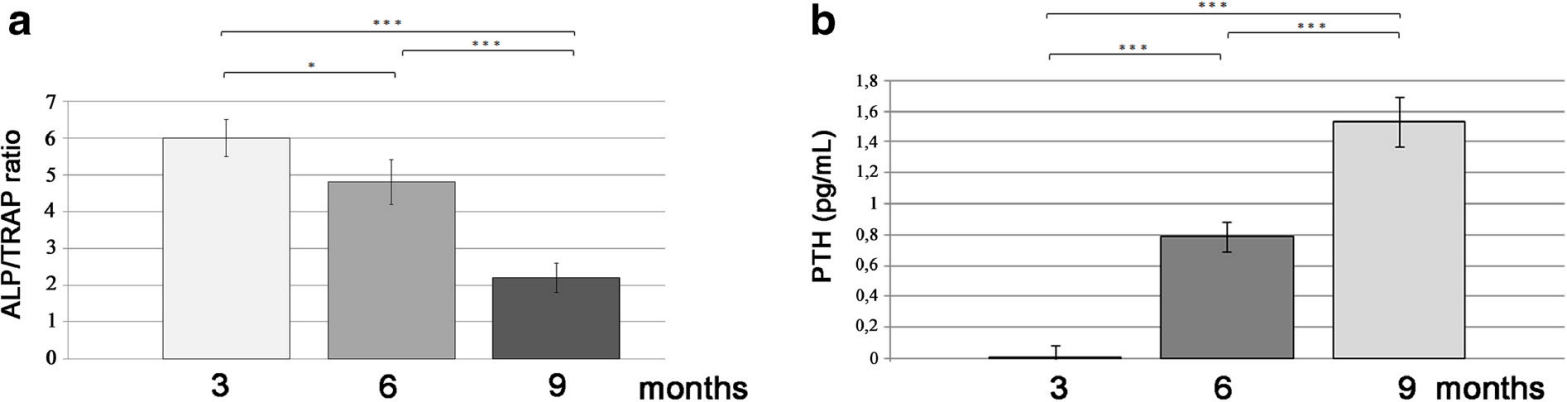
**a** Calculated ALP/TRAP ratio. A trend to bone resorption has been indicated by the lowering of ALP/TRAP ratio as the age increases (3 months vs 6 months, *p* < 0.05; 3 months vs 9 months, *p* < 0.001; 6 months vs 9 months, *p* < 0.001). **b** *PTH level measured in fish blood* showed that the hormone significantly increased with age (3 months vs 6 months, *p* < 0.001; 3 months vs 9 months, *p* < 0.001; 6 months vs 9 months, *p* < 0.001)

To evaluate the bone turnover activity in aging scales, ALP/TRAP ratio was calculated showing a progressive reduction from 3- to 9-month-old fish with a decrease of 20% in 6-month-old fish and of 63% in 9-month-old fish (Fig. 4a; 3 months vs 6 months, *p* < 0.05; 3 months vs 9 months, *p* < 0.001; 6 months vs 9 months, *p* < 0.001). These data clearly indicated an increasing tendency to bone resorption in old scales. The calcium metabolism and its hormonal regulation network play an important role in human bone turnover; an example is the parathormone (PTH) pathway [13]. To investigate whether the age modulates the hormonal signalling involved in zebrafish bone metabolism*,* PTH has been quantified in the blood of 3-, 6 -and 9-month-old fish. The results indicated that PTH significantly increased with age in fish blood (Fig. 4b; 3 months vs 6 months, *p* < 0.001; 3 months vs 9 months, *p* < 0.001; 6 months vs 9 months, *p* < 0.001).

### Prednisolone treatment–induced bone resorption activity in aged fish

**Fig. 5:**
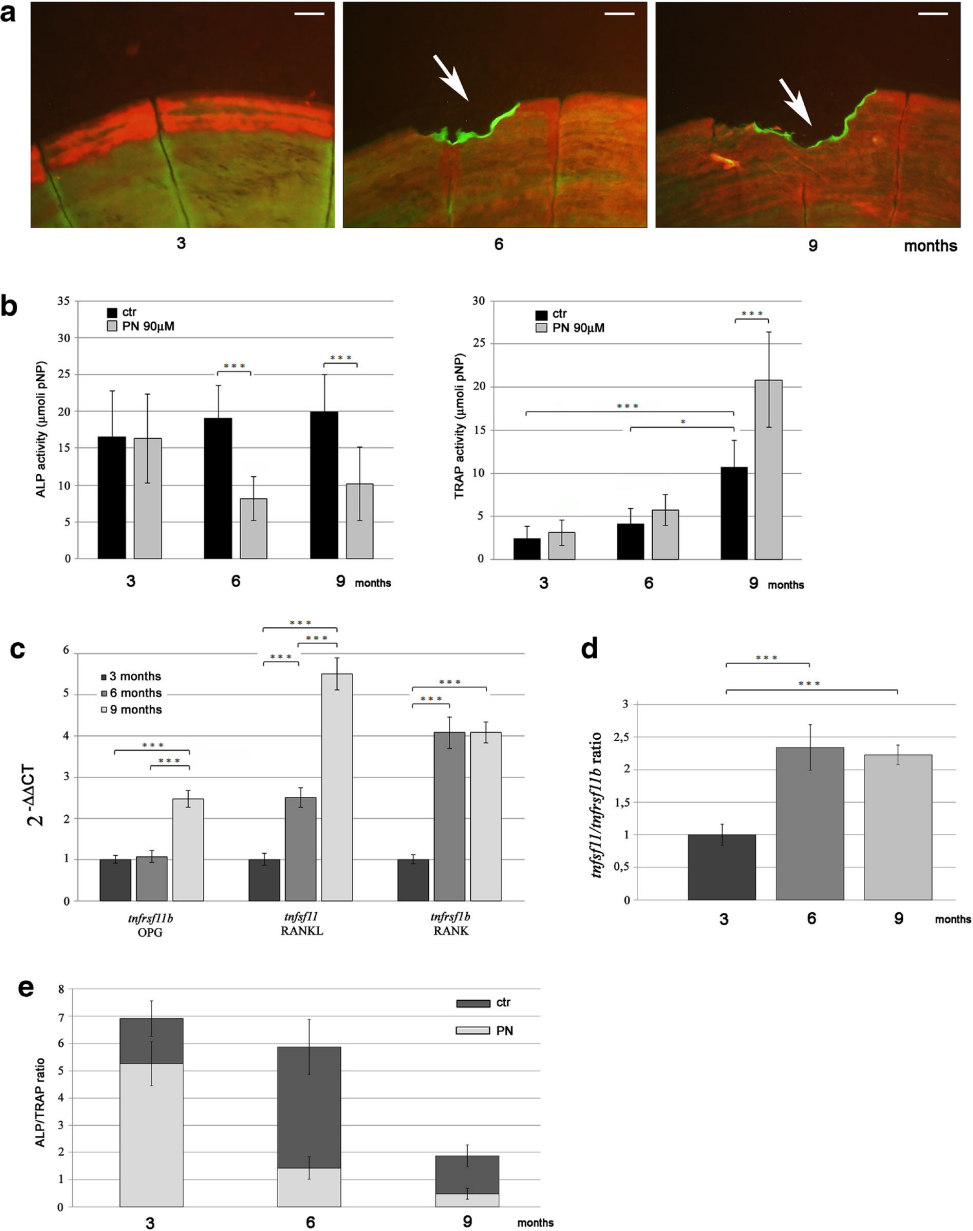
**a** Alizarin red/calcein live double staining of PN-treated scales from fish of different ages (scale bar = 0.1 mm). PN inhibited the formation of the new mineralized scale ring in fish of all ages and induced a significant bone resorption in 6- and 9-month-old fish scales (white arrows). **b** ALP and TRAP activity assay on PN-treated scales from fish of different ages. A significant reduction of ALP activity has been highlighted in 6- and 9-month-old fish treated with PN compared with untreated fish of the same age. The TRAP activity was significantly increased in PN-treated 9-month-old fish compared with earlier ages as well as with untreated fish of the same age (ALP: 6 months CTR vs 6 months PN, *p* < 0.001; 9 months CTR vs 9 months PN, *p* < 0.001; TRAP: 3 months CTR vs 9 months CTR, *p* < 0.001; 6 months CTR vs 9 months CTR, *p* < 0.05; 9 months CTR vs 9 months PN, *p* < 0.001). **c** Gene expression analysis for bone marker genes on PN-treated zebrafish scales. The expression of *tnfrsf1b*, *tnfsf11* and *tnfrsf11b* (homologs of human *rank*, *rankl* and *opg*, respectively) was found increased in all ages after PN treatment (OPG: 3 months vs 9 months, *p* < 0.001; 6 months vs 9 months, *p* < 0.001. RANKL: 3 months vs 6 months, *p* < 0.001; 3 months vs 9 months, *p* < 0.001; 6 months vs 9 months, *p* < 0.001. RANK: 3 months vs 6 months, *p* < 0.001; 3 months vs 9 months, *p* < 0.001). **d** Calculated *tnfsf11*/*tnfrsf11b* (*rankl/opg*) ratio. The upregulation of *tnfsf11* (human *rankl)* allowed the increase of the *tnfsf11*/*tnfrsf11b* (*rankl/opg*) ratio in 6- and 9 month-old PN-treated fish (3 months vs 6 months, *p* < 0.001; 3 months vs 9 months, *p* < 0.001). **e** ALP/TRAP ratio in PN-treated fish. Compared with the untreated fish, ALP/TRAP ratio decreased more rapidly during aging after PN treatment (6 months CTR vs 3 months CTR, − 15%, *p* < 0.05; 9 months CTR vs 3 months CTR, − 73%, *p* < 0.001; 9 months CTR vs 6 months CTR, − 68%, *p* < 0.001; 6 months PN vs 3 months PN, − 73%, *p* < 0.001; 9 months PN vs 3 months PN, − 90%, bp < 0.001; 9 months PN vs 6 months PN, − 18%, *p* < 0.01)

Prednisolone (PN) is a glucocorticoid that is well known to be a pro-osteoporotic agent in humans [1] as well as in fish [10, 41]. To investigate whether PN exerts different effects on fish bone at different age, we treated 3-, 6- and 9-month-old fish with 80 μM PN for 2 weeks. After that, the mineralization rate and the presence of resorption areas have been evaluated in the scales of treated and non-treated fish. Control fish have been maintained in E3 medium only since our preliminary studies demonstrated that treating in E3 medium with DMSO at the same concentration used to perform PN treatment does not affect fish health status and scale metabolism (data not shown). The analysis has shown that PN treatment inhibited the deposition of the new mineralized ring in all ages and induced a significant bone resorption in 6- and 9-month-old fish scales (Fig. 5a). In particular, the scale area characterized by bone loss was more extensive in 9-month-old (− 3.8%) than in 6-month-old (− 1.8%) fish (Table 1).

**Table 1:** Catabolic characteristics of aging scales of untreated (ctr) and PN-treated (PN) fish

Months	3		6		9	
%	RA	TPS	RA	TPS	RA	TPS
ctr	0	0	0	0	− 1.02	63
PN	0	0	− 1.81	70	− 3.8	95

Resorption area (RA) was expressed as percentage of scale surface lost, while the intensity of the resorbing stimulus was expressed as percentage of TRAP-positive scales (TPS)

Scales from 3-, 6- and 9-month-old PN-treated fish have been also used to measure ALP activity through biochemical assay. A significant reduction of ALP activity has been highlighted in 6 and 9 months-old fish treated with PN compared with untreated fish of the same age (Fig. 5b left panel; ALP: 6 months CTR vs 6 months PN, *p* < 0.001; 9 months CTR vs 9 months PN, *p* < 0.001).

The biochemical analysis of TRAP activity performed on scales explanted from the same fish has indicated a significant increase in PN-treated 9-month-old fish compared with earlier ages as well as with untreated fish of the same age (Fig. 5b right panel; TRAP: 3 months CTR vs 9 months CTR, *p* < 0.001; 6 months CTR vs 9 months CTR, *p* < 0.05; 9 months CTR vs 9 months PN, *p* < 0.001). The number of TRAP-positive scales also increased with age and after PN treatment (Table 1).

The study of the gene expression performed by real-time PCR highlighted an age-dependent increase of the *tnfrsf1b*, *tnfsf11* and *tnfrsf11b* (homologs of human *rank*, *rankl* and *opg*, respectively) in scales of PN-treated 3-, 6- and 9-month-old fish (Fig. 5c; OPG: 3 months vs 9 months, *p* < 0.001; 6 months vs 9 months, *p* < 0.001. RANKL: 3 months vs 6 months, *p* < 0.001; 3 months vs 9 months, *p* < 0.001; 6 months vs 9 months, *p* < 0.001. RANK: 3 months vs 6 months, *p* < 0.001; 3 months vs 9 months, *p* < 0.001). Among these, a stronger upregulation of *tnfsf11* (homolog of human *rankl)* in 6- and 9-month-old PN-treated fish allowed to increase the *tnfsf11*/*tnfrsf11b* (*rankl/opg*) ratio (Fig. 5d; 3 months vs6 months, *p* < 0.001; 3 months vs 9 months, *p* < 0.001).

As consequence, the ALP/TRAP ratio in PN-treated fish decreased more rapidly during aging compared with untreated fish (Fig. 5e).

## Discussion

Several studies demonstrated a clear relationship between aging and bone metabolism in vertebrates, in which early phases associated with changes at molecular and cellular level play a crucial role [2, 17]. Due to the complexity of the regulation systems, the mechanisms are not yet completely understood. Bone markers, systemic metabolic signals, cell behaviour and gene expression could be better investigated in a simple animal model to dissect in detail the early phase of bone aging. Zebrafish is a powerful animal model with many advantages such as small size, low cost of maintenance and rapid development. In addition, thanks to his high conservation of bone structure, it is an excellent model of osteogenesis, bone metabolism and bone remodelling [54]. In particular, the scale represents an optimum model for bone metabolism studies at cellular and molecular level [39, 42]. In the present work, we investigated the early mechanisms of bone aging in zebrafish using the scale as innovative read-out system. Adult fish from 3 to 9 months have been used based on previous studies about age-dependent vertebral microalterations in adult zebrafish [6, 38, 46].

The analysis of the scale ring demonstrated that the osteodeposition rate decreases with age from 3 months to terminate at 9 months with a complete arrest of its growth. These data suggested that the osteodeposition process in fish scale is regulated during the life and coordinated with the body growth, as well as in humans.

In zebrafish scale, the cells responsible for the initial bone matrix deposition are characterized by intense ALP activity [40] as well as in humans [23]. The histological staining in old-fish scales highlighted an age-dependent downregulation of ALP signal in the matrix-producing cells, suggesting that their activity is progressively switched off during aging, whereas the total ALP activity did not change significantly in the whole scale. In fact, the variation of ALP signal in few matrix-deposing cells (black arrows in Fig. 2b) is clearly too low to be detected with biochemical ALP activity measurement in whole scale.

Since ALP is expressed in the early differentiation process of the human osteoblasts [18], we hypothesize that the shutdown of the osteodeposition activity is probably due to an impairment of the differentiation processes. To support these data, in humans, age-related bone loss is predominantly due to a reduced osteoblast activity [11] and associated with a decrease in the number of MSCs and their differentiation potential [31, 56].

The analysis of bone catabolic activity, measured in fish scales through the specific biomarker TRAP, indicates a statistically significant increase related to fish age. In 9-month-old fish, we observed 63% of TRAP-positive scales (Table 1), in which a visible bone resorption process takes place along the scale border.

Osteoclasts and TRAP activity are modulated by human bone aging processes since it has been demonstrated that osteoclast cells become more active on aged bone matrix than on young one [8, 22].

The increase of TRAP activity in aged fish scales could be explained mainly by the upregulation of the *tnfrsf1b* (homolog of human *rank*) in 6- and 9-month-old fish. It is known that age exerts multiple effects on expression of gene coding receptors and endogenous bone regulatory factors. In particular, age-related upregulation of RANK may indicate an increase in the number osteoclast progenitors and/or the number of receptors per cell. It has been also demonstrated in mice that such condition could make osteoclast progenitors more responsive to M-CSF and to RANKL [4]. The increase of TRAP activity allows, consequently, to a progressive decline in the ALP/TRAP ratio during aging in fish scales.

The biochemical changes associated to aging in fish involved also the PTH blood level, which increases progressively with age. This condition is found also in humans where sex steroids, calcium and renal function may induce an increase of PTH levels the in blood of aged women and men [25]. The increased serum PTH may act directly on osteoclastic activity increasing bone resorption as demonstrated in the fracture-healing model of orchiectomized mice [30]. The mechanism of PTH-induced osteoclast differentiation and activation could be mediated by parathormone receptor 1 (PTHR1) and Vacuolar-type H^+^-ATPase (V-ATPase), both expressed in osteoclast precursors [32]. However, we should also consider that environment and diet are different in human and fish.

We conclude that zebrafish possesses a form of senile osteoporosis detectable in the scales and characterized by biochemical, histological and molecular evidences similar to aged human bone. Since we used male animals, the oestrogen hormones were not involved in the regulation of bone metabolism in our model.

The scales, as part of the dermal skeleton, are not subjected to mechanical or gravitational forces, and, in addition, they show great similarity with human bone in terms of cell function and biochemical regulations [40, 47]. For these reasons, the scale represents an excellent model to study the basic mechanisms of metabolic changes in aging bone.

The glucocorticoid PN is a well-known pro-osteoporotic agent in humans [28] as well as in zebrafish [10, 15, 41].

The treatment of fish with PN inhibits the formation of the new scale ring in fish of all ages and, in addition, induces an important osteoporosis-like phenotype in the scales of older fish. The absence of matrix deposition in young animals indicates that PN has important negative effects on osteoblast activity as confirmed by the reduction of ALP activity after glucocorticoid treatment in the scales of the same age. The bone resorption stimulation of PN can be explained by the enhanced TRAP activity in PN-treated scales with respect to untreated fish of the same age. Indeed, the resorption area is proportional to the TRAP activity level at 6- and 9-month-old fish.

It has been demonstrated that the incidence of vertebral fractures are age-dependent in human patients treated with high-dose of glucocorticoids [33, 49]. Our data support this evidence, suggesting that, in humans as well in fish, these pro-osteoporotic drugs are more effective on old bone than younger one.

The increase of osteoclast activity may be due to a PN-dependent over-stimulation of *tnfsf11* (homolog of human *rankl*) in 6- and 9-month-old fish, which causes the elevation of *tnfsf11*/*tnfrsf11b* (*rankl/opg*) ratio. These data suggest that PN stimulates osteoclast activity, enhancing the catabolic stimuli, which are already elevated in the old bone due to the upregulation of RANK (Table 2).

**Table 2:** Summary of bone alterations in aged zebrafish scales from untreated and PN-treated fish

PN	RANK/OPG	RANK	ALP/TRAP	BONE LOSS
–	–	↑	↓	↑
+	↑	↑	↓↓	↑↑

Physiologically, elevated RANK is associated to old zebrafish scales, whereas pro-osteoporotic stimuli, such as PN, enhance RANKL expression, which over-stimulates TRAP-dependent resorption activity in osteoclasts

Although not performed on proteins, the gene expression analysis of OPG, RANKL and OPG/RANKL ratio can be informative in zebrafish as demonstrated in dexamethasone-treated model [34] and in adult scales after biophysical stimulation [26].

Talking about molecular mechanisms, it has been demonstrated that glucocorticoid treatment promotes osteoclast differentiation and activity increasing RANKL production [27] In vitro, RANKL has been found to induce cell-cell fusion during osteoclast differentiation and syncytial organization [36]. Our data indicated that a similar mechanism may be used by PN in zebrafish scale.

In conclusion, the present work demonstrated for the first time that zebrafish develops a form of bone aging detectable in the scales where the osteoblasts become progressively inactive from 6 to 9 months and the osteoclasts are activated at 9 months. These data confirm that early crucial events of bone aging in zebrafish should be studied from 3 to 9 months. In fact, previous works on zebrafish have demonstrated that structural microalterations were detected in spine between 6 and 9 months [6, 38, 46], as well as in male rodent models, where the early events of age-related osteopenia have been detected around few months ([20]., [24]).

**Fig. 6:**
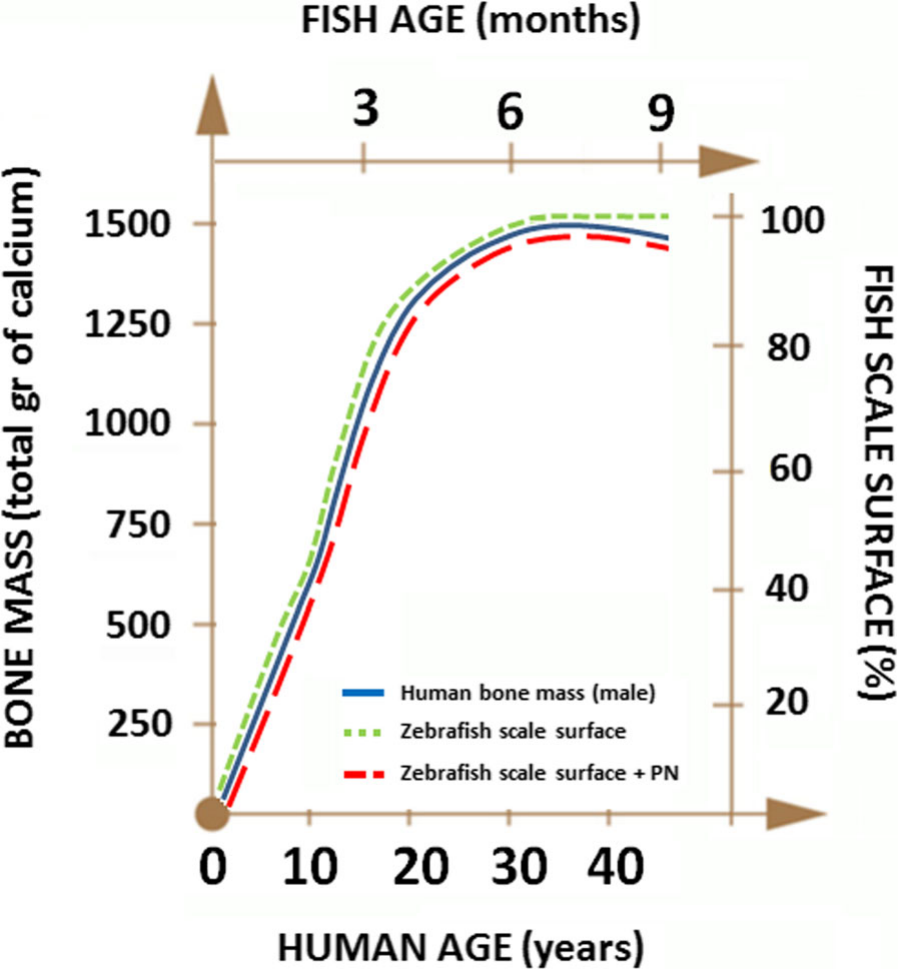
Comparison between bone growth (human male) and scale growth (zebrafish male) at different age. In zebrafish, the catabolic phase is accelerated by prednisolone (PN)

Interestingly, these data indicates that fish scale and male human skeleton share a very similar growth curve in which osteodeposition rate is maximal in juvenile stage (< 6 months/30 years) and slightly declines in early adult stage (6–9 months/30–45 years), generating a negative bone balance (Fig. 6).

Senile male osteoporosis in humans is associate with a reduction in BMD between 0.5 and 1% per year [9], while in female is reported between 2 and 4% per year in the first 5 to 10 years after menopause [29]. Considering the differences between humans and fish, we can assume that a reduction of 1.02% in fish scale surface could be considered a model of senile male osteoporosis comparable with that of humans.

In aged male fish, the bone resorption activity in the scale of adult fish is accelerated when treated with pro-osteoporotic agents like prednisolone (Fig. 6). In fact, also in humans, osteoclast activity is higher in aged bones [22].

The whole life span of fish does not have linear timeline with respect to humans because of phylogenetic distance, like rodents [12, 45]. Nevertheless, considering that *Danio rerio* reproductive phase spans, more or less, between 3 mpf (human 12–14 years, adolescence) and 12 mpf (human 55 years), the curve of bone mass seems to be similar in fish and humans. Further studies will be addressed to verify the persistence and any modulation of bone loss phenotype in the scales of older fish (> 1 year). This study will better elucidate the mechanisms of the clinical complications in the late phase of age-dependent bone loss.

In conclusion, despite the anatomical and phylogenetic distance, the model of senile male osteoporosis in zebrafish scale can help to understand the early mechanisms of the physiological bone aging and screen potential new anti-osteoporotic drugs.

## References

[1] Amiche MA, Albaum JM, TadroussM PP, Lévesque LE, Adachi JD, Cadarette SM (2016). Fracture risk in oral glucocorticoid users: a Bayesian meta-regression leveraging control arms of osteoporosis clinical trials. Osteoporos Int.

[2] Boskey AL, Imbert L (2017). Bone quality changes associated with aging and disease: a review. Ann N Y Acad Sci.

[3] Busse B, Galloway JL, Gray RS, Harris MP, Kwon RY (2020). Zebrafish: an emerging model for orthopedic research. J Orthop Res.

[4] Cao J, Venton L, Sakata T, Halloran BP (2003). Expression of RANKL and OPG correlates with age-related bone loss in male C57BL/6 mice. J Bone Miner Res.

[5] Carnovali M, Mariotti M, Banfi G (2016). The adult zebrafish as polyhedric model for skeletal studies. J Biol Regul Homeost Agents.

[6] Chang Z, Chen PY, Chuang YJ, Akhtar RJ (2018). Zebrafish as a model to study bone maturation: nanoscale structural and mechanical characterization of age-related changes in the zebrafish vertebral column. Mech Behav Biomed Mater.

[7] Cheng M, Zhuang J, Li TT, Li WJ, Chen Y, Xu LZ (2015). Age will affect the growth and mineralization ability of the rat osteoblast. Clin Lab.

[8] Chung PL, Zhou S, Eslami B, Shen L, LeBoff MS, Glowacki J (2014). Effect of age on regulation of human osteoclast differentiation. J Cell Biochem.

[9] D'Amelio P, Isaia GC (2015). Male osteoporosis in the elderly. Int J Endocrinol.

[10] de Vrieze E, van Kessel MA, Peters HM, Spanings FA, Flik G, Metz JR (2014). Prednisolone induces osteoporosis-like phenotype in regenerating zebrafish scales. Osteoporos Int.

[11] Duque G, Troen BR (2008). Understanding the mechanisms of senile osteoporosis: new facts for a major geriatric syndrome. J Am Geriatr Soc.

[12] Dutta S, Sengupta P (2016). Men and mice: relating their ages. Life Sci.

[13] Fatayerji D, Eastell R (1999). Age-related changes in bone turnover in men. J Bone Miner Res.

[14] Fiedler IAK, Schmidt FN, Wölfel EM, Plumeyer C, Milovanovic P, Gioia R, Tonelli F, Bale HA, Jähn K, Besio R, Forlino A, Busse B (2018). Severely impaired bone material quality in Chihuahua Zebrafish resembles classical dominant human osteogenesis imperfecta. J Bone Miner Res.

[15] Geurtzen K, Vernet A, Freidin A, Rauner M, Hofbauer LC, Schneider JE, Brand M, Knopf F (2017). Immune suppressive and bone inhibitory effects of prednisolone in growing and regenerating Zebrafish tissues. J Bone Miner Res.

[16] Gibon E, Lu L, Goodman SB (2016). Aging, inflammation, stem cells, and bone healing. Stem Cell Res Ther.

[17] Goltzman D (2019). The aging skeleton. Adv Exp Med Biol.

[18] Golub EE, Boesze-Battaglia K (2007). The role of alkaline phosphatase in mineralization. Curr Opin Orthop.

[19] Guggenbuhl P (2009). Osteoporosis in males and females: is there really a difference?. Joint Bone Spine.

[20] Halloran BP, Ferguson VL, Simske SJ, Burghardt A, Venton LL, Majumdar S (2002). Changes in bone structure and mass with advancing age in the male C57BL/6J mouse. J Bone Miner Res.

[21] Hayes AJ, Reynolds S, Nowell MA, Meakin LB, Habicher J, Ledin J, Bashford A, Caterson B (2013). Changes to their vertebrae that resemble osteoarthritis. PLoS One.

[22] Henriksen K, Leeming DJ, Byrjalsen I, Nielsen RH, Sorensen MG, Dziegiel MH, Martin TJ, Christiansen C, Qvist P, Karsdal MA (2007). Osteoclasts prefer aged bone. Osteoporos Int.

[23] Hoemann CD, El-Gabalawy H, McKee MD (2009). In vitro osteogenesis assays: influence of the primary cell source on alkaline phosphatase activity and mineralization. Pathol Biol (Paris).

[24] Jilka RL (2013). The relevance of mouse models for investigating age-related bone loss in humans. J Gerontol A Biol Sci Med Sci.

[25] Kennel KA, Riggs BL, Achenbach SJ, Oberg AL, Khosla S (2003). Role of parathyroid hormone in mediating age-related changes in bone resorption in men. Osteoporos Int.

[26] Kitamura K, Takahira K, Inari M, Satoh Y, Hayakawa K, Tabuchi Y, Ogai K, Nishiuchi T, Kondo T, Mikuni-Takagaki Y, Chen W, Hattori A, Suzuki N (2013). Zebrafish scales respond differently to in vitro dynamic and static acceleration: analysis of interaction between osteoblasts and osteoclasts. Comp Biochem Physiol A.

[27] Komori T (2016). Glucocorticoid signaling and bone biology. Horm Metab Res.

[28] Leib ES, Winzenrieth R (2016). Bone status in glucocorticoid-treated men and women. Osteoporos Int.

[29] Levis S, Altman R (1998). Bone densitometry: clinical considerations. Arthritis Rheum.

[30] Li W, Yuan L, Tong G, He Y, Meng Y, Hao S, Chen J, Guo J, Bringhurst R, Yang D (2018). Phospholipase C signaling activated by parathyroid hormone mediates the rapid osteoclastogenesis in the fracture healing of orchiectomized mice. BMC Musculoskelet Disord.

[31] Liu H, Xia X, Li B (2015). Mesenchymal stem cell aging: mechanisms and influences on skeletal and non-skeletal tissues. Exp Biol Med (Maywood).

[32] Liu S, Zhu W, Li S, Ma J, Zhang H, Li Z, Zhang L, Zhang B, Li Z, Liang X, Shi W (2016). Bovine parathyroid hormone enhances osteoclast bone resorption by modulating V-ATPase through PTH1R. Int J Mol Med.

[33] Liu Y, Dimango E, Bucovsky M, Agarwal S, Nishiyama K, Guo XE, Shane E, Stein EM (2018). Abnormal microarchitecture and stiffness in postmenopausal women using chronic inhaled glucocorticoids. Osteoporos Int.

[34] Lu SY, Wang CY, Jin Y, Meng Q, Liu Q, Liu ZH, Liu KX, Sun HJ, Liu MZ (2017). The osteogenesis-promoting effects of alpha-lipoic acid against glucocorticoid-induced osteoporosis through the NOX4, NF-kappaB, JNK and PI3K/AKT pathways. Sci Rep.

[35] Manolagas SC (2010). From estrogen-centric to aging and oxidative stress: a revised perspective of the pathogenesis of osteoporosis. Endocr Rev.

[36] Miyamoto T (2011). Regulators of osteoclast differentiation and cell-cell fusion. Keio J Med.

[37] Mank JE (2007). The evolution of sexually selected traits and antagonistic androgen expression in actinopterygiian fishes. Am Nat.

[38] Monma Y, Shimada Y, Nakayama H, Zang L, Nishimura N, Tanaka T (2019). Aging-associated microstructural deterioration of vertebra in zebrafish. Bone Rep.

[39] Pasqualetti S, Banfi G, Mariotti M (2012). Osteoblast and osteoclast behaviour in zebrafish cultured scales. Cell Tissue Res.

[40] Pasqualetti S, Banfi G, Mariotti M (2012). The zebrafish scale as model to study the bone mineralization process. J Mol Histol.

[41] Pasqualetti S, Congiu T, Banfi G, Mariotti M (2015). Alendronate rescued osteoporotic phenotype in a model of glucocorticoid-induced osteoporosis in adult zebrafish scale. Int J Exp Pathol.

[42] Perrson P, Takagi Y, Björnsson BT (1995). Tartrate resistant acid phosphatases as a marker for scale resorption in rainbow trout, Oncorhynchus mykiss: effects of estradiol-17β treatment and refeeding. Fish Physiol Biochem.

[43] Porcelli T, Maffezzoni F, Pezzaioli LC, Delbarba A, Cappelli C, Ferlin A. Management of endocrine disease: male osteoporosis: diagnosis and management—should the treatment and target be the same as for female osteoporosis? Eur J Endocrinol. 2020. 10.1530/eje-20-0034. 10.1530/EJE-20-0034 32544873

[44] Roy B, Curtis ME, Fears LS, Nahashon SN, Fentress HM (2016). Molecular mechanisms of obesity-induced osteoporosis and muscle atrophy. Front Physiol.

[45] Sengupta P (2013). The laboratory rat: relating its age with human’s. Int J Prev Med.

[46] Shanthanagouda AH, Guo BS, Ye RR, Chao L, Chiang MW, Singaram G, Cheung NK, Zhang G, Au DW (2014). Japanese medaka: a non-mammalian vertebrate model for studying sex and age-related bone metabolism in vivo. PLoS One.

[47] Sire JY, Akimenko MA (2004). Scale development in fish: a review, with description of sonic hedgehog (shh) expression in the zebrafish (Danio rerio). Int J Dev Biol.

[48] Suzuki N, Danks JA, Maruyama Y, Ikegame M, Sasayama Y, Hattori A, Nakamura M, Tabata MJ, Yamamoto T, Furuya R, Saijoh K, Mishima H, Srivastav AK, Furusawa Y, Kondo T, Tabuchi Y, Takasaki I, Chowdhury VS, Hayakawa K, Martin TJ (2011). Parathyroid hormone 1 (1–34) acts on the scales and involves calcium metabolism in goldfish. Bone.

[49] Tatsuno I, Sugiyama T, Suzuki S, Yoshida T, Tanaka T, Sueishi M, Saito Y (2009). Age dependence of early symptomatic vertebral fracture with high-dose glucocorticoid treatment for collagen vascular diseases. J Clin Endocrinol Metab.

[50] Tomecka MJ, Ethiraj LP, Sánchez LM, Roehl HH, Carney TJ (2019). Clinical pathologies of bone fracture modelled in zebrafish. Dis Model Mech.

[51] Wang L, Banu J, McMahan CA, Kalu DN (2001). Male rodent model of age-related bone loss in men. Bone.

[52] Wei Y, Sun Y (2018). Aging of the bone. Adv Exp Med Biol.

[53] Westerfield M (2007). The zebrafish book: a guide for the laboratory use of zebrafish (*Danio rerio*).

[54] Witten PE, Huysseune A (2009). A comparative view on mechanisms and functions of skeletal remodeling in teleost fish, with special emphasis on osteoclasts and their function. Biol Rev Camb Philos Soc.

[55] Zanatta M, Valenti MT, Donatelli L, Zucal C, Dalle Carbonare L (2012). Runx-2 gene expression is associated with age-related changes of bone mineral density in the healthy young-adult population. J Bone Miner Metab.

[56] Zhou S, Greenberger JS, Epperly MW, Goff JP, Adler C, Leboff MS, Glowacki J (2008). Age-related intrinsic changes in human bone-marrow-derived mesenchymal stem cells and their differentiation to osteoblasts. Aging Cell.

